# Association of Hospital Quality and Neighborhood Deprivation With Mortality After Inpatient Surgery Among Medicare Beneficiaries

**DOI:** 10.1001/jamanetworkopen.2022.53620

**Published:** 2023-01-30

**Authors:** Adrian Diaz, Stacy Tessler Lindau, Samilia Obeng-Gyasi, Justin B. Dimick, John W. Scott, Andrew M. Ibrahim

**Affiliations:** 1 Department of Surgery, The Ohio State University, Columbus; 2 Department of Surgery, University of Michigan, Ann Arbor; 3 Center for Healthcare Outcomes and Policy, University of Michigan, Ann Arbor; 4 Department of Obstetrics & Gynecology, University of Chicago, Chicago, Illinois; 5 Department of Medicine–Geriatrics, University of Chicago, Chicago, Illinois; 6 Taubman College of Architecture & Urban Planning, University of Michigan, Ann Arbor

## Abstract

**Importance:**

Although the hospital at which a patient is treated is a known source of variation in mortality after inpatient surgery, far less is known about how the neighborhoods from which patients come may also contribute.

**Objective:**

To compare postoperative mortality among Medicare beneficiaries based on the level of neighborhood deprivation where they live and hospital quality where they received care.

**Design, Setting, and Participants:**

This cross-sectional study examined outcomes among Medicare beneficiaries undergoing 1 of 5 common surgical procedures (colon resection, coronary artery bypass, cholecystectomy, appendectomy, or incisional hernia repair) between 2014 and 2018. Hospital quality was assigned using the Centers for Medicare & Medicaid Services Star Rating. Each beneficiary’s neighborhood was identified at the census tract level and sorted in quintiles based on its Area Deprivation Index score, a composite measure of neighborhood quality, including education, employment, and housing quality. A risk matrix across hospital quality and neighborhood deprivation was created to determine the relative contribution of each to mortality after surgery. Data were analyzed from June 1 to December 31, 2021.

**Exposures:**

Hospital quality and neighborhood deprivation.

**Main Outcomes and Measures:**

The main outcome was risk-adjusted 30-day mortality after surgery using a multivariable logistic regression model taking into account patient factors and procedure type.

**Results:**

A total of 1 898 829 Medicare beneficiaries (mean [SD] age, 74.8 [7.0] years; 961 216 [50.6%] male beneficiaries; 28 432 [1.5%] Asian, 145 160 [77%] Black, and 1 622 304 [86.5%] White beneficiaries) were included in analyses. Patients from all neighborhood deprivation group quintiles sought care at hospitals across hospital quality levels. For example, 9.1% of patients from the highest deprivation neighborhoods went to a hospital in the highest star rating of quality and 4.2% of patients from the lowest deprivation neighborhoods went to a hospital in the lowest star rating of quality. Thirty-day risk-adjusted mortality varied across high- and low-quality hospitals (4.3% vs 7.2%; adjusted odds ratio [aOR], 1.78; 95% CI, 1.66-1.92) and across the least and most deprived neighborhoods (4.5% vs 6.8%; aOR, 1.58; 95% CI, 1.53-1.64). When combined, comparing patients from the least deprived neighborhoods going to high-quality hospitals vs patients from the most deprived neighborhoods going to low-quality hospitals, the variation increased further (3.8% vs 8.1%; aOR, 2.20; 95% CI, 1.96-2.46).

**Conclusions and Relevance:**

These findings suggest that characteristics of a patient’s neighborhood and the hospital where they received treatment were both associated with risk of death after commonly performed inpatient surgical procedures. The associations of these factors on mortality may be additive. Efforts and investments to address variation in postoperative mortality should include both hospital quality improvement as well as addressing drivers of neighborhood deprivation.

## Key Points

**Question:**

Is postoperative mortality among Medicare beneficiaries associated with the level of neighborhood deprivation of where they live and quality of hospital where they receive care?

**Findings:**

In this cross-sectional review of 1 898 829 Medicare beneficiaries undergoing 1 of 5 common surgical procedures, patients from the least deprived neighborhoods going to high-quality hospitals had a 3.9% probability of postoperative mortality compared with 8.1% among patients from the most deprived neighborhoods going to low-quality hospitals, a significant difference.

**Meaning:**

These findings suggest that the scope of hospital-driven efforts and investments to minimize disparities in postoperative mortality should include attention to factors associated with socioeconomic deprivation in the communities where patients live.

## Teaser

This cross-sectional study compares postoperative mortality among Medicare beneficiaries based on the level of neighborhood deprivation where they live and hospital quality where they received care.

## Introduction

Rates of death following inpatient surgery vary widely across both the hospitals at which patients receive care and the neighborhoods in which they live.^1,2^ Widespread and resource-intensive efforts to reduce variation in surgical mortality have primarily focused on hospital quality improvement. For example, the American College of Surgeons National Quality Improvement Program^3^ provides participating hospitals a risk-adjusted, outcomes-based program to measure and improve the quality of surgical care. Alternatively, pay-for-performance plans, such as the Surgical Care Improvement Project of the Centers for Medicare & Medicaid Services (CMS), aims at creating incentives for greater adherence to evidence-based practices for perioperative care.^4^ Because neither of these large-scale efforts have been able to reduce variation in mortality^5,6^ and surgery-related spending now accounts for more than half of CMS’s annual budget,^7^ there is increasing interest from surgeons, health systems, and health insurers to understand and intervene on factors outside the hospital, including the neighborhood living conditions of people undergoing surgery.^8,9^

While there is evolving evidence that structural and systemic factors concentrated in socioeconomically deprived neighborhoods are associated with worse outcomes and earlier death for people with chronic medical conditions,^10,11,12,13,14^ far less is known about the associations for individuals with conditions requiring hospital-based surgical intervention. Previous studies have suggested multiple possible mechanisms by which individuals who receive surgical care who are from underresourced neighborhoods experience worst outcomes.^15,16,17^ First, individuals in these neighborhoods may disproportionately receive care at lower-quality, lower-resourced hospitals due to geographic proximity of these neighborhoods to lower-quality hospitals and limited information about where to receive best care and other barriers to access.^18^ Second, high rates of chronic illnesses cluster in lower-income neighborhoods and, as a result of barriers to health care, are more likely to go undiagnosed. These underlying conditions may be exacerbated by the physiologic hardship and psychosocial stress of undergoing a surgical procedure, thereby contributing to worse postoperative outcomes.^16^ To our knowledge, however, no studies have incorporated both the deprivation level of a patient’s neighborhood and the quality of hospital where patients receive care to understand the relative associations of each factor with postoperative mortality.

In that context, we sought to evaluate the relative association of surgical outcomes with neighborhood deprivation and hospital quality using a large national cohort of Medicare beneficiaries who had undergone common inpatient surgical procedures. We hypothesized that death after inpatient surgery would vary widely by both hospital quality and neighborhood deprivation. Moreover, we also hypothesized that these associations would be additive.

## Methods

This study was deemed exempt from review and informed consent by the University of Michigan institutional review board because data were deidentified. This study is reported following the Strengthening the Reporting of Observational Studies in Epidemiology (STROBE) reporting guideline.

### Data Source and Population

Data from 100% of claims in the Medicare Provider Analysis and Review (MedPAR) file for calendar years 2014 to 2018 at nonfederal acute care hospitals were used for this study. This data set included 5 years of the most recently available data across a selection of surgical procedures that are commonly performed across the United States. Procedure codes for colon resection, coronary artery bypass, cholecystectomy, appendectomy, and incisional hernia repair from the *International Classification of Diseases, Ninth Revision* (*ICD-9*) and *International Statistical Classification of Diseases and Related Health Problems, Tenth Revision* (*ICD-10*) Procedure Coding System from the MedPAR file, with confirmatory Current Procedural Terminology codes from the Medicare Carrier File, were used to define the cohort. These specific procedures have been commonly used for evaluating surgical quality among Medicare beneficiaries (eTable 3 in Supplement 1).^5,19^ Hospital identifiers from the MedPAR file were linked to the American Hospital Association Annual Survey for each corresponding year, which provided hospital characteristics. Patient race and ethnicity were self-reported as Asian, Black, Hispanic, White, or other (includes North American Native or other as reported by the ResDac Data dictionary for the data used). Race and ethnicity were included in analyses to control for confounding.

### Assessment of Neighborhood Deprivation

Current neighborhood characteristics for each included patient were measured using the Area Deprivation Index (ADI) at the 9-digit zip code level.^20,21^ The ADI is a validated, neighborhood-level composite index reflecting 17 dimensions captured in the American Community Survey and US Census Survey data. The ADI national rankings range from 1 to 100, with least disadvantaged neighborhood conditions designated by lower scores and most disadvantaged by higher scores. Examples of neighborhood-level factors used to generate the ADI include education, employment, housing quality, and poverty measures. Neighborhoods were rank ordered by the ADI and divided into ordinal quintiles. Lowest deprivation neighborhoods were defined as those in the quintile with the lowest ADI. Similarly, those in the quintile with the highest ADI rates were labeled highest deprivation neighborhoods. ADI Version 3 (2018) was used to coincide with the patient level data used for this study.

### Assessment of Hospital Quality

Hospital rating was assessed by the Centers for Medicare & Medicaid Services (CMS) Overall Hospital Quality Star Rating program.^22^ This rating program attempts to provide a single public measure of hospital quality, summarizing dozens of metrics on Hospital Compare. Of note, 30-day postoperative mortality is not one of the metrics used to calculate ratings. We calculated baseline hospital-level performance (scores, ranks, and star categories) for the April 2021 ratings using the methods used by CMS.^5^ Hospital Star Rating was selected as a measure of hospital quality because the measure is publicly available and designed to be used by patients and referring physicians to select sites of care and by payers and purchasers to direct beneficiaries, select hospitals for contracts, and set hospital payments.

### Statistical Analysis

The overall goal of this analysis was to understand the risk of mortality after common surgical procedures taking into account both neighborhood deprivation and hospital quality. Postoperative mortality was assessed by 30-day postoperative mortality rates from 2 sources. First, mortality in the hospital was determined by vital status at the time of discharge. Additionally, the Medicare Beneficiary Denominator File was used to ascertain any mortality occurring within 30 days of discharge from the index operation, including patients who died after discharge from their index admission or after transfer to another facility. This definition of 30-day mortality is an established quality measure for surgical care.^23,24^ Because hospitals vary in the patient populations for whom they care and the distribution of procedures they perform, the rates of 30-day mortality needed to be risk-adjusted. Using a well-established risk-adjustment strategy for surgical outcomes described by Elixhauser et al^25^ and Southern et al,^26^ a multivariable logistic regression model that accounted for patient age, sex, comorbidities (as described by Elixhauser et al; each comorbidity as an indicator variable), operation type (ie, appendectomy, cholecystectomy, coronary artery bypass graft, colectomy, or incisional hernia repair), and operation acuity (ie, elective, urgent, or emergency) was used to calculate a risk-adjusted rate of 30-day mortality for each hospital. Our model also included year of operation as a categorical variable to account for secular trends. Finally, the analysis was adjusted for *ICD-9* and *ICD-10* principal diagnosis and procedure codes (as indicator variables) to capture differences in diagnosis and surgical approach. To account for hospital characteristics, hospital bed size, teaching status, profit status, and staffing ratios were included in the model. To adjust for clustering within hospitals, robust standard errors were used for all models. To test for possible patient nesting bias, 2 different hierarchical models were created. First, to test nesting within hospitals, a 2-tier hierarchical model was created using patient factors (level 1) and hospital random effects (level 2).

We also performed a reliability adjustment to account for random variation in observed outcomes. Because hospitals vary in their case volumes, it may be difficult to determine if the observed outcomes at low-volume centers truly reflect the quality they provide or statistical noise (ie, chance). Our approach to reliability adjustment that accounts for statistical noise has been previously described.^27,28^ Briefly, this technique uses hierarchical modeling and empirical Bayes estimates to shrink lower-volume hospitals toward the overall population mean proportionally to the strength of their statistical signal (ie, their surgical volume). Thus, our final quintiles to identify high- and low-quality hospitals were based on risk- and reliability-adjusted outcomes.

After identifying high- and low-deprivation neighborhoods and high- and low-quality hospitals, we compared them with respect to patient and hospital characteristics using χ^2^ and Wilcoxon rank sum tests as appropriate. Based on the same multivariable logistic regression model, the probability of risk-adjusted mortality was calculated for each patient. We then sought to describe the interaction of neighborhood deprivation and hospital rating on postoperative 30-day mortality risk. To do so, we compared risk-adjusted 30-day mortality rates across both quintiles of neighborhoods and hospitals.

All analyses were performed using Stata statistical software version 16 (StataCorp). All tests were 2-sided, and significance was determined by *P* < .05. Data were analyzed from June 1 to December 31, 2021.

## Results

Between 2014 and 2018, Medicare beneficiaries residing in 1 569 184 unique neighborhoods underwent 1 898 829 operations (mean [SD] age, 74.8 [7.0] years; 961 216 [50.6%] male beneficiaries): 327 545 beneficiaries (17.3%) resided in neighborhoods in the first quintile of deprivation (ie, with the lowest deprivation), 423 194 beneficiaries (22.3%) resided in neighborhoods in the second quintile, 435 763 beneficiaries (23.0%) resided in neighborhoods in the third quintile, 391 671 beneficiaries (20.6%) resided in neighborhoods in the fourth quintile, and 320 656 beneficiaries (16.9%) resided in neighborhoods in the fifth quintile (ie, with the highest deprivation). According to self-report, 28 432 beneficiaries (1.5%) were Asian, 145 160 beneficiaries (7.7%) were Black, 38 551 beneficiaries (2.0%) were Hispanic, and 1 622 304 beneficiaries (86.5%) were White. Distribution of patient age and sex characteristics were relatively similar across neighborhoods (Table 1). However, the proportion of Black beneficiaries was lowest (14 993 individuals [4.7%]) in the lowest deprivation quintile and highest (54 062 individuals [17.0%]) in neighborhoods in the highest quintile of deprivation (*P* < .001). The proportion of operative procedures that were elective was significantly higher in the lowest deprivation neighborhoods (141 046 procedures [43.1%]) than in the highest deprivation neighborhoods (131 094 procedures [40.9%]; *P* < .001). The number of Elixhauser comorbidities was lowest among patients from the lowest deprivation neighborhoods, with a mean (SD) of 3.0 (1.9) comorbidities, and increased along each quintile to 3.4 (2.0) comorbidities for patients in neighborhoods that had the highest deprivation (*P* < .001) (Table 1).

**Table 1.** Patient Characteristics by Neighborhood Area Deprivation Quintile

Characteristic	Patients, No. (%)						*P* value
	Overall (N = 1 898 829)	Neighborhood deprivation quintile^a^					
		First (n = 327 545)	Second (n = 423 194)	Third (n = 435 763)	Fourth (n = 391 671)	Fifth (n = 320 656)	
Age, mean (SD), y	74.8 (7.0)	75.3 (7.2)	74.8 (7.0)	74.7 (7.0)	74.7 (7.0)	74.4 (7.0)	<.001
Sex							
Male	961 216 (50.6)	169 994 (51.9)	218 619 (51.7)	222 298 (51.0)	195 531 (49.9)	154 774 (48.3)	<.001
Female	937 613 (49.4)	157 551 (48.1)	204 575 (48.3)	213 465 (49.0)	196 140 (50.1)	165 882 (51.7)	<.001
Race and ethnicity^b^							
Asian	28 432 (1.5)	14 668 (4.5)	6784 (1.6)	3512 (0.8)	2155 (0.6)	1313 (0.4)	<.001
Black	145 160 (7.7)	14 993 (4.7)	20 960 (5.0)	24 600 (5.7)	30 545 (7.9)	54 062 (17.0)	<.001
Hispanic	38 551 (2.0)	7589 (2.3)	8919 (2.1)	7049 (1.6)	6937 (1.8)	8057 (2.5)	<.001
White	1 622 304 (86.5)	269 699 (84.0)	371 636 (89.1)	389 136 (90.3)	342 899 (88.3)	248 934 (78.1)	<.001
Other	64 382 (3.4)	20 596 (6.3)	14 895 (3.5)	11 466 (2.6)	9135 (2.3)	8290 (2.6)	<.001
Surgical procedure							
Appendectomy	133 834 (7.0)	27 862 (8.5)	31 652 (7.5)	29 702 (6.8)	24 973 (6.4)	19 645 (6.1)	<.001
Coronary artery bypass grafting	405 082 (21.3)	62 173 (19.0)	88 248 (20.9)	95 553 (21.9)	87 676 (22.4)	71 432 (22.3)	
Cholecystectomy	505 041 (26.6)	86 227 (26.3)	111 814 (26.4)	115 778 (26.6)	104 442 (26.7)	86 780 (27.1)	
Colectomy	579 495 (30.5)	103 603 (31.6)	129 849 (30.7)	131 393 (30.2)	118 282 (30.2)	96 368 (30.1)	
Incisional hernia	275 377 (14.5)	47 680 (14.6)	61 631 (14.6)	63 337 (14.5)	56 298 (14.4)	46 431 (14.5)	
Elective admission	809 485 (42.6)	141 046 (43.1)	182 610 (43.2)	188 031 (43.1)	166 704 (42.6)	131 094 (40.9)	<.001
Comorbidities							
Mean (SD)	3.2 (1.9)	3.0 (1.9)	3.1 (1.9)	3.2 (1.9)	3.3 (1.9)	3.4 (2.0)	<.001
Congestive heart failure	189 700 (10.0)	27 979 (8.5)	38 865 (9.2)	43 232 (9.9)	42 115 (10.8)	37 509 (11.7)	<.001
Valvular disease	102 136 (5.4)	19 844 (6.1)	23 679 (5.6)	23 380 (5.4)	20 143 (5.1)	15 090 (4.7)	<.001
Pulmonary circulation disease	27 067 (1.4)	4613 (1.4)	6136 (1.4)	6054 (1.4)	5650 (1.4)	4614 (1.4)	.10
Peripheral vascular disease	201 870 (10.6)	35 875 (11.0)	42 658 (10.1)	45 355 (10.4)	42 173 (10.8)	35 809 (11.2)	<.001
Paralysis	36 379 (1.9)	5948 (1.8)	7323 (1.7)	7939 (1.8)	7652 (2.0)	7517 (2.3)	<.001
Other neurological disorders	127 956 (6.7)	20 339 (6.2)	27 924 (6.6)	29 635 (6.8)	27 284 (7.0)	22 774 (7.1)	<.001
Chronic pulmonary disease	400 344 (21.1)	54 631 (16.7)	81 265 (19.2)	92 856 (21.3)	91 433 (23.3)	80 159 (25.0)	<.001
Diabetes, no chronic complications	378 192 (19.9)	53 623 (16.4)	77 787 (18.4)	87 246 (20.0)	83 959 (21.4)	75 577 (23.6)	<.001
Diabetes, chronic complications	238 334 (12.6)	36 824 (11.2)	49 690 (11.7)	53 684 (12.3)	51 744 (13.2)	46 392 (14.5)	<.001
Hypothyroidism	310 806 (16.4)	52 186 (15.9)	69 998 (16.5)	73 161 (16.8)	64 996 (16.6)	50 465 (15.7)	<.001
Kidney failure	308 416 (16.2)	48 730 (14.9)	64 975 (15.4)	70 476 (16.2)	66 135 (16.9)	58 100 (18.1)	<.001
Liver disease	84 831 (4.5)	15 795 (4.8)	18 945 (4.5)	19 005 (4.4)	16 659 (4.3)	14 427 (4.5)	<.001
Peptic ulcer disease with bleeding	18 099 (1.0)	3015 (0.9)	3874 (0.9)	4116 (0.9)	3877 (1.0)	3217 (1.0)	<.001
AIDS	785 (0.0)	195 (0.1)	160 (0.0)	149 (0.0)	125 (0.0)	156 (0.0)	<.001
Lymphoma	13 875 (0.7)	2931 (0.9)	3272 (0.8)	3169 (0.7)	2557 (0.7)	1946 (0.6)	<.001
Metastatic cancer	99 872 (5.3)	19 160 (5.8)	22 316 (5.3)	22 185 (5.1)	19 952 (5.1)	16 259 (5.1)	<.001
Solid tumor without metastasis	61 757 (3.3)	11 271 (3.4)	13 574 (3.2)	13 789 (3.2)	12 490 (3.2)	10 633 (3.3)	<.001
Rheumatoid arthritis	60 457 (3.2)	10 134 (3.1)	13 858 (3.3)	14 210 (3.3)	12 570 (3.2)	9685 (3.0)	<.001
Coagulopathy	187 313 (9.9)	32 269 (9.9)	42 356 (10.0)	43 288 (9.9)	38 101 (9.7)	31 299 (9.8)	<.001
Obesity	359 249 (18.9)	50 700 (15.5)	77 734 (18.4)	85 777 (19.7)	79 054 (20.2)	65 984 (20.6)	<.001
Weight loss	187 720 (9.9)	30 754 (9.4)	39 386 (9.3)	42 176 (9.7)	39 696 (10.1)	35 708 (11.1)	<.001
Fluid and electrolyte disorders	668 423 (35.2)	107 174 (32.7)	146 152 (34.5)	153 688 (35.3)	141 214 (36.1)	120 195 (37.5)	<.001
Chronic blood loss anemia	35 833 (1.9)	5804 (1.8)	7421 (1.8)	7976 (1.8)	7632 (1.9)	7000 (2.2)	<.001
Deficiency anemias	347 745 (18.3)	58 680 (17.9)	73 908 (17.5)	76 794 (17.6)	72 803 (18.6)	65 560 (20.4)	<.001
Alcohol abuse	31 127 (1.6)	5419 (1.7)	7012 (1.7)	6945 (1.6)	6183 (1.6)	5568 (1.7)	<.001
Drug abuse	9614 (0.5)	1784 (0.5)	2100 (0.5)	2056 (0.5)	1916 (0.5)	1758 (0.5)	<.001
Psychoses	32 453 (1.7)	6650 (2.0)	7024 (1.7)	6947 (1.6)	6232 (1.6)	5600 (1.7)	<.001
Depression	182 152 (9.6)	27 620 (8.4)	40 597 (9.6)	43 779 (10.0)	39 595 (10.1)	30 561 (9.5)	<.001
Hypertension	1 385 190 (72.9)	227 787 (69.5)	304 640 (72.0)	318 786 (73.2)	290 983 (74.3)	242 994 (75.8)	<.001

^a^ Neighborhood deprivation is measured using the Area Deprivation Index the first quintile indicating the least disadvantaged neighborhood conditions and the fifth quintile, most disadvantaged neighborhood conditions. Examples of neighborhood-level factors incorporated within the Area Deprivation Index include education, employment, housing quality, and poverty measures.

^b^ Race and ethnicity are self-reported by beneficiaries to Social Security Administration, which is then reported to Medicare. Beneficiaries can only choose 1 option. The other category includes North American Native or other as reported by the ResDac Data dictionary for the data used.

Hospitals across CMS star ratings varied by structural characteristics. Nurse-to-bed ratio increased from 1.9 at 1-star hospitals to 2.5 at 5-star hospitals (*P* < .001). Bed size and teaching status did not vary consistently across star ratings (eTable 1 in Supplement 1). Furthermore, the mean (SD) neighborhood ADI score of patients treated at a given hospital decreased from 55.4 (27.4) at the 1-star hospitals to 41.8 (26.3) at 5-star hospitals (*P* < .001). Similarly, the mean (SD) neighborhood ADI of hospitals decreased from 69.9 (21.6) at the 1-star hospitals to 50.4 (26.3) at the 5-star hospitals (*P* < .001).

**Figure 1.**
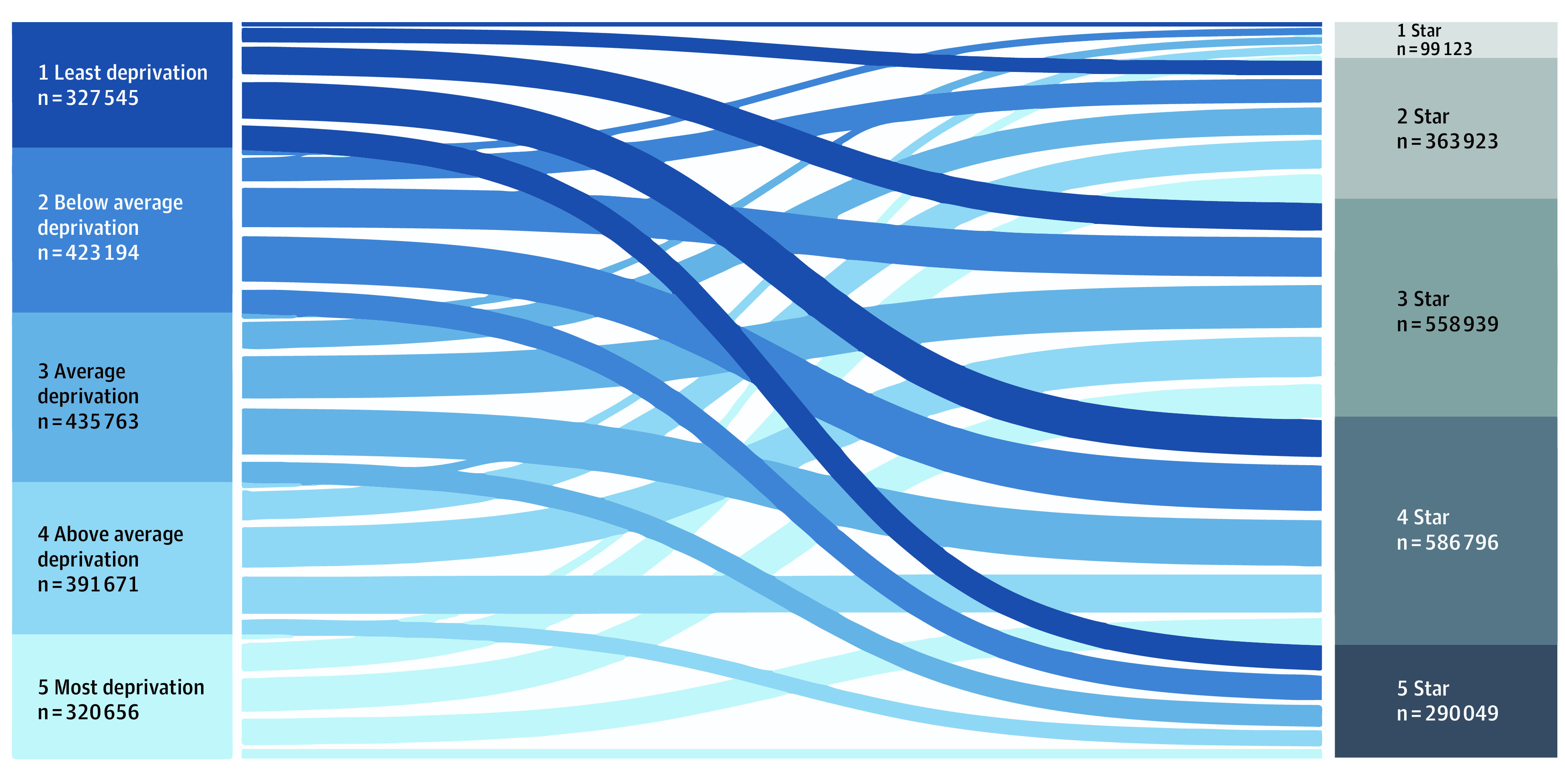
Sankey Flow Diagram of Patient Neighborhood Quality and Location of Care

The distribution of patients from neighborhoods to hospitals is shown in Figure 1. There was no clear pattern of patient sorting from any given neighborhood to any given hospital according to hospital quality. For example, 9.1% of patients from the highest deprivation neighborhoods went to a hospital with the highest star rating and 4.2% of patients from the lowest deprivation neighborhoods went to a hospital with the lowest star rating (Figure 1).

**Figure 2.**
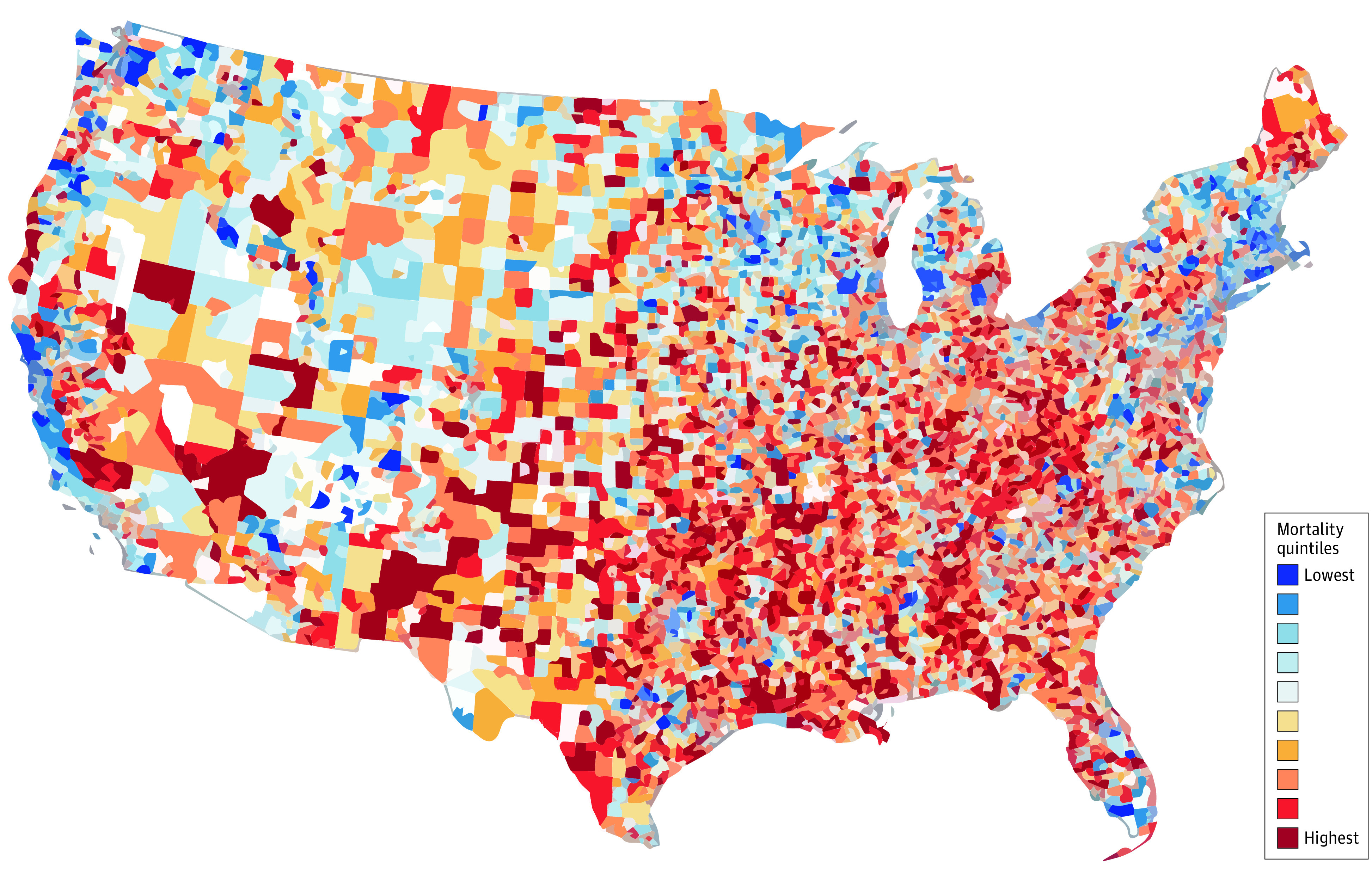
Variation in Postoperative Mortality Across US Neighborhoods Although analysis was conducted at the 9-digit zip code level, we visually display them here at the 5-digit zip-code level for adherence with the Data Use Agreement of Medicare Data.

Overall, postoperative risk-adjusted mortality varied widely by neighborhood ADI (Figure 2). The risk-adjusted 30-day mortality rate was 4.5% (95% CI, 4.4%-4.7%) in the lowest deprivation neighborhoods (first quintile; reference group) and displayed a significant stepwise increase with each quintile of increasing deprivation (eFigure 1 in Supplement 1): 5.1% (95% CI, 5.0%-5.2%; aOR, 1.13; 95% CI, 1.10-1.17) for the second deprivation quintile, 5.5% (95% CI, 5.4%-5.6%; aOR, 1.26; 95% CI, 1.22-1.30) for the third deprivation quintile, 6.1% (95% CI, 6.0%-6.2%; aOR, 1.41; 95% CI, 1.36-1.45) for the fourth deprivation quintile, and 6.8% (95% CI, 6.7%-6.9%; aOR, 1.58; 95% CI, 1.53-1.64) for the fifth (highest) deprivation quintile. A similar stepwise increase was observed across CMS star ratings (eFigure 1 in Supplement 1), with the 5-star hospitals (reference group) having a risk-adjusted mortality of 4.3% (95% CI, 4.2%-4.5%), then 5.1% (95% CI, 4.9%-5.2%; aOR, 1.19; 95% CI, 1.14-1.24) in 4-star hospitals, 5.9% (95% CI, 5.8%-6.0%; aOR, 1.41; 95% CI, 1.35-1.48) at 3-star hospitals, 6.6% (95% CI, 6.4%-6.8%; aOR, 1.60; 95% CI, 1.53-1.69) at 2-star hospitals, and 7.2% (95% CI, 6.9%-7.7%; aOR, 1.78; 95% CI, 1.66-1.92) at 1-star hospitals.

**Figure 3.**
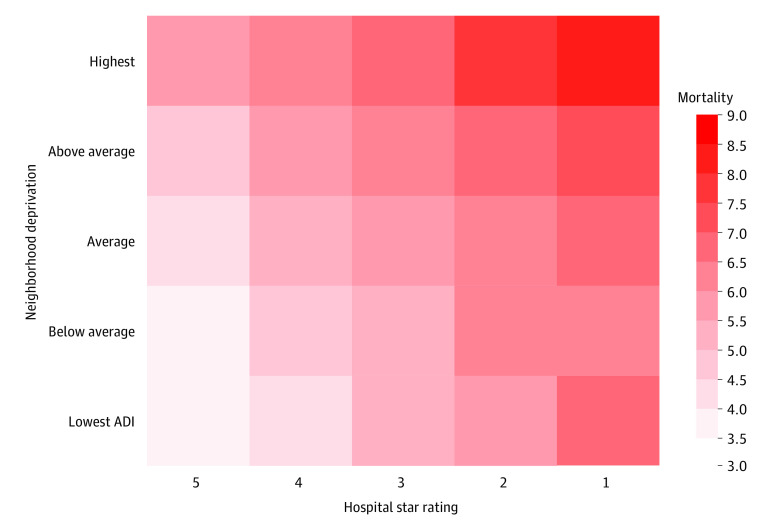
Risk-Matrix of Surgical Mortality by Hospital Quality and Neighborhood Deprivation Risk-adjusted rates were derived from marginal means in logistic regression models. All models were adjusted for patient age, sex, and 27 Elixhauser comorbidities, type of admission (ie, elective, nonelective), overall time trends, hospital characteristics, and patient’s Area Deprivation Index. The Area Deprivation Index rankings range from 1 to 100, with least disadvantaged neighborhood conditions designated by lower scores and most disadvantaged by higher scores. Examples of neighborhood-level factors incorporated within the Area Deprivation Index include education, employment, housing quality, and poverty measures.

When combined, the beneficiary neighborhood and hospital star rating further increased variation in 30-day risk-adjusted mortality (Figure 3). Specifically, beneficiaries from the lowest deprivation neighborhoods undergoing their operation at 5-star hospitals had a risk-adjusted 30-day mortality of 3.8% (95% CI, 3.6%-3.9%). Conversely, risk-adjusted 30-day mortality among beneficiaries from the highest deprivation neighborhoods undergoing their operation at 1-star hospitals was 8.1% (95% CI, 7.7%-8.4%), demonstrating an absolute difference of 4.3 (95% CI, 3.3-4.6) percentage points (aOR, 2.20; 95% CI, 1.96-2.46). Of note, these patterns persisted for both Black and White beneficiaries; however, Black beneficiaries had overall higher rates of mortality (eFigure 2 in Supplement 1). Furthermore, there was significant variation in 30-day mortality across quintiles of both neighborhood deprivation and hospital quality (Table 2). For example, among beneficiaries residing in a neighborhood with deprivation in the third quintile, there was an absolute mortality difference of 2.2 (95% CI, 1.9-2.5) percentage points at 1-star compared with 5-star hospitals (aOR, 1.68; 95% CI, 1.59-1.78). Similarly, for beneficiaries who underwent their operation at a 3-star hospital, there was an absolute mortality difference of 1.9 (95% CI, 1.7-2.0) percentage points for beneficiaries residing in the highest deprivation neighborhoods compared with those in the lowest deprivation neighborhoods (aOR, 1.45; 95% CI, 1.40-1.50). Our sensitivity analyses using neighborhood and hospital hierarchical models taking into account how patients may nest demonstrated similar point estimates to our main analyses using robust standard errors (eTable 2 in Supplement 1).

**Table 2.** Risk-Adjusted 30-Day Mortality Across Hospital Quality and Neighborhood Area Deprivation Quintiles

Factor	Mortality^a^		Absolute difference, percentage points	aOR (95% CI)^b^
	1-Star hospital	5-Star hospital		
ADI quintile^c^				
First	6.7 (6.5-7.0)	3.8 (3.6-3.9)	3.0 (2.6-3.4)	1.94 (1.78-2.12)
Second	6.4(6.2-6.6)	3.9 (3.8-4.0)	2.5 (2.2-2.8)	1.82 (1.70-1.94)
Third	6.7 (6.5-6.9)	4.5 (4.3-4.6)	2.2 (1.9-2.5)	1.68 (1.59-1.78)
Fourth	7.4 (7.2-7.7)	5.0 (4.8-5.1)	2.5 (2.1-2.8)	1.67 (1.56-1.79)
Fifth	8.1(7.7-8.4)	5.7 (5.4-5.9)	2.4 (1.9-2.9)	1.60 (1.46-1.75)
Hospital stars	Highest ADI^c^	Lowest ADI^c^		
1	8.1 (7.7-8.4)	6.7 (6.5-7.0)	1.3 (0.86-1.8)	1.28 (1.18-1.38)
2	7.6 (7.4-7.8)	5.8 (5.6-5.9)	1.8 (1.6-2.1)	1.39 (1.32-1.47)
3	6.9 (6.8-7.0)	5.1 (5.0-5.2)	1.9 (1.7- 2.0)	1.45 (1.40-1.50)
4	6.2 (6.0-6.3)	4.2 (4.1-4.3)	2.0 (1.8-2.1)	1.54 (1.48-1.61)
5	5.7 (5.4-5.9)	3.8 (3.6-3.9)	1.9 (1.6–2.2)	1.55 (1.45-1.66)

Abbreviations: ADI, Area Deprivation Index; aOR, adjusted odds ratio.

^a^ Risk-adjusted rates were derived from marginal means in logistic regression models. All models adjusted for patient age, sex, and 27 Elixhauser comorbidities, type of admission (ie, elective, non-elective), year of operation, hospital characteristics, and patients Area Deprivation Index.

^b^ Reference groups for odds ratios are the highest-rated hospitals and lowest ADI neighborhoods.

^c^ The ADI rankings range from 1 to 100, with the first quintile indicating the least disadvantaged neighborhood conditions and fifth quintile indicating most disadvantaged neighborhood conditions. Examples of neighborhood-level factors incorporated within the ADI include education, employment, housing quality, and poverty measures.

## Discussion

This cross-sectional study had 3 principal findings that improve understanding of variation in postoperative surgical mortality. First, despite longstanding efforts in mitigating variation in surgical mortality across hospitals, significant variation persists. Second, we found significant variation in surgical mortality among patients from across neighborhoods at each level of hospital quality. Finally, neighborhood deprivation and hospital quality may have an additive association with postoperative mortality. Specifically, beneficiaries from the neighborhoods with the highest deprivation who underwent surgery at the lowest-quality hospitals had 2.8-fold greater odds of postoperative mortality compared with their counterparts from the neighborhoods with the lowest deprivation undergoing surgery at the highest-quality hospitals. Taken together, these findings suggest that the neighborhoods people come from and the hospitals they go are both associated with risk of death after commonly performed inpatient surgical procedures.

Prior efforts to understand hospital-level variation have focused on structural and process elements of care. For example, staffing ratios, timely recognition and management of complications, and surgery-specific volumes have all been identified as sources of variation.^1,23,29^ This study reinforces those findings in identifying continued hospital-level variation in outcomes and extends understanding of this variation by attributing previously unaccounted variation to the area deprivation level of the neighborhoods where patients undergoing surgery live. Importantly, the current study challenges the notion that patients from more deprived neighborhoods simply go to low-quality hospitals. Specifically, at least 1 in 10 beneficiaries from each neighborhood quintile obtained care at each of the 5 quintiles of hospital quality. Although star ratings are meant for public use, little is known about how patients use them. Lack of knowledge about hospital quality ratings and other factors, like trust and stigma, may explain why patients from all neighborhoods obtained their care at hospitals with varying quality.^30^

Understanding the role of the built environment and other neighborhood conditions on health care outcomes has largely focused on chronic and medical conditions. Allostatic load—a measure of cumulative physiological stress due to chronic adverse socioenvironmental stressors—has been proposed as a mechanism through which adverse socioenvironmental stressors exert their influence on health outcomes.^31^ The ADI, a possible surrogate of allostatic load, has been associated with higher rates of diabetes, cardiovascular disease, and other diseases; increased utilization of health services; and earlier death.^10,11,12,13,14^ Our study found similar associations between neighborhood-level socioeconomic deprivation and postoperative mortality. Moreover, our findings also identified hospital quality as an additive factor associated with worse outcomes.

The findings of this cross-sectional study have several implications for health care and public policy stakeholders. First, these findings support community benefit and other policies designed to incentivize hospital investment in and examination of the role they play in advancing structural equity in the communities they serve.^32^ In addition, driven in part by value-based payment policies, a growing number of health care systems are piloting programs to identify patients living in neighborhoods with higher risk of poor surgical outcomes with interventions to optimize their health before surgery.^33^ Second, for insurers considering value-based payment models, these findings suggest that community-level structural disadvantages may need to be accounted for to appropriately evaluate a hospital’s quality of care.^34,35^ Third, for government leaders and policy makers dedicated to improving health care delivery and equity, these findings support aligning both hospital- and neighborhood-level policies as a strategy to improve health care outcomes. The National Academies of Sciences, Engineering, and Medicine advocate for this kind of alignment as a key structural-level strategy for achieving integrated medical and social care.^36^

### Limitations

This study has some limitations. First, because only Medicare patients were studied, our results may not be entirely generalizable to the US population. However, Medicare is the only national data set with granularity to identify a patient’s hospital of care and neighborhood residence so that both exposures can be evaluated. Moreover, policies aimed at neighborhood interventions are most likely to originate with CMS, which has already piloted such programs.^8^ Therefore, using Medicare data to query these associations may increase relevance of the study findings to key policy makers. The study is also limited by our inability to identify an exact mechanism by which neighborhood deprivation may drive worse surgical outcomes. However, we did identify a robust dose-response association between postoperative mortality and neighborhood deprivation—a finding we observed across procedures—suggesting a meaningful association between the exposure and outcome. Next, data on beneficiary ADI was extrapolated from each beneficiary’s 9-digit zip code and not from a patient-level variable. However, the 9-digit zip code is the most granular geographic area available from Medicare, and typically consists of approximately 1 city block. The 9-digit zip code is comparable to census block groups, the smallest geographic area for which the Bureau of the Census collects. Furthermore, as with other studies using administrative data, the findings of this study are subject to residual confounding due to unmeasured factors, such as noncoded comorbidities. Use of mortality as the primary outcome helps to mitigate the potential of confounding. Mortality is less susceptible to coding biases than other outcome measures (eg, postoperative complications) because Medicare compiles this outcome from multiple sources.^15^ We also applied several modeling strategies, including logistic regression with robust standard errors and hierarchical modeling, to demonstrate the robustness of our results.

## Conclusions

This cross-sectional study’s findings suggest that characteristics of a patient’s neighborhood and the hospital where they received treatment were both associated with risk of death after commonly performed inpatient surgical procedures. The associations of these factors on mortality may be additive. Efforts and investments to address variation in postoperative mortality should include both hospital quality improvement as well as addressing drivers of neighborhood deprivation.

## Notes

Supplement 1. **eTable 1.** Hospital Characteristics by Hospital Star Ratings **eFigure 1.** Variation in Surgical Mortality Across Hospitals and Neighborhoods **eFigure 2.** Risk Matrix of 30-day Mortality After Inpatient Surgery by Neighborhood Deprivation and Hospital Quality Among Black and White Medicare Beneficiaries **eTable 2.** Comparison of 30-Day Mortality Using Multivariable Logistic Regression Model With Clustering vs 2-Level and 3-Level Hierarchical Models **eTable 3.** List of *ICD-9* and *ICD-10* Procedure Codes

Supplement 2.
Data Sharing Statement
