# Supplementary material for: Association of Hospital Quality and Neighborhood Deprivation With Mortality After Inpatient Surgery Among Medicare Beneficiaries
Source: JAMA Netw Open. 2023 Jan 30;6(1):e2253620. doi: 10.1001/jamanetworkopen.2022.53620 (PMC9887494; doi:10.1001/jamanetworkopen.2022.53620)
Supplement: Supplement 1. — eTable 1. Hospital Characteristics by Hospital Star Ratings eFigure 1. Variation in Surgical Mortality Across Hospitals and Neighborhoods eFigure 2. Risk Matrix of 30-day Mortality After Inpatient Surgery by Neighborhood Deprivation and Hospital Quality Among Black and White Medicare Beneficiaries eTable 2. Comparison of 30-Day Mortality Using Multivariable Logistic Regression Model With Clustering vs 2-Level and 3-Level Hierarchical Models eTable 3. List of ICD-9 and ICD-10 Procedure Codes [file jamanetwopen-e2253620-s001.pdf]

Supplemental Online Content

Diaz A, Lindau ST, Obeng-Gyasi S, Dimick JB, Scott JW, Ibrahim AM. Association of hospital quality and neighborhood deprivation with mortality after inpatient surgery among Medicare beneficiaries. *JAMA Netw Open*. 2023;6(1):e2253620. doi:10.1001/jamanetworkopen.2022.53620

eTable 1. Hospital Characteristics by Hospital Star Ratings
eFigure 1. Variation in Surgical Mortality Across Hospitals and Neighborhoods
eFigure 2. Risk Matrix of 30-day Mortality After Inpatient Surgery by Neighborhood Deprivation and Hospital Quality Among Black and White Medicare Beneficiaries
eTable 2. Comparison of 30-Day Mortality Using Multivariable Logistic Regression Model With Clustering vs 2-Level and 3-Level Hierarchical Models
eTable 3. List of *ICD-9* and *ICD-10* Procedure Codes

This supplemental material has been provided by the authors to give readers additional information about their work.

© 2023 Diaz A et al. *JAMA Network Open.*

eTable 1. Hospital Characteristics by Hospital Star Ratings

		Hospital Stars					
	Overall N=1,898,829	One N=99,122 (5.2)	Two N=363,923 (19.2)	Three N=558,939 (29.4)	Four N=586,796 (30.9)	Five N=290,049 (15.3)	p-value
No. of Hospitals	3,240	200 (6.2)	667 (20.6)	983 (30.3)	953 (29.4)	437 (13.5)	--
Mean ADI, Hospital	61.8 (23.1)	69.9 (21.6)	67.8 (21.5)	62.8 (21.8)	59.7 (22.6)	50.4 (26.3)	<0.001
Mean ADI, Patient	50.0 (27.0)	55.4 (27.4)	56.3 (26.6)	52.0 (26.6)	47.3 (26.6)	41.8 (26.3)	<0.001
ADI							
Lowest	327,545 (17.2%)	13,642 (13.8%)	41,525 (11.4%)	83,884 (15.0%)	113,318 (19.3%)	75,176 (25.9%)	
Below Average	423,194 (22.3%)	19,027 (19.2%)	70,719 (19.4%)	119,542 (21.4%)	138,639 (23.6%)	75,267 (25.9%)	
Average	435,763 (22.9%)	20,294 (20.5%)	80,543 (22.1%)	130,625 (23.4%)	140,185 (23.9%)	64,116 (22.1%)	
Above Average	391,671 (20.6%)	22,831 (23.0%)	85,958 (23.6%)	122,815 (22.0%)	113,915 (19.4%)	46,152 (15.9%)	
Highest	320,656 (16.9%)	23,328 (23.5%)	85,178 (23.4%)	102,073 (18.3%)	80,739 (13.8%)	29,338 (10.1%)	
Region							
Mid-west	432,190 (22.8)	8,179 (8.3)	54,780 (15.1)	106,374 (19.1)	156,362 (26.7)	106,495 (36.8)	<0.001
North-East	325,408 (17.2)	21,196 (21.6)	62,788 (17.3)	91,761 (16.5)	106,525 (18.2)	43,138 (14.9)	
South	777,166 (41.1)	50,997 (51.9)	190,136 (52.4)	247,988 (44.6)	219,648 (37.5)	68,397 (23.6)	
West	357,460 (18.9)	17,885 (18.2)	55,123 (15.2)	110,239 (19.8)	102,626 (17.5)	71,587 (24.7)	
Urban	1,742,731 (92.1)	87,549 (89.1)	330,886 (91.2)	513,892 (92.4)	532,566 (91.0)	277,838 (95.9)	<0.001
Teaching Hospital	386,846 (20.4)	20,431 (20.8)	61,330 (16.9)	99,070 (17.8)	132,655 (22.7)	73,360 (25.3)	<0.001
Mean Nurse: Bed Ratio, (SD)	2.1 (0.8)	1.9 (0.7)	1.9 (0.9)	2.0 (0.6)	2.2 (0.7)	2.5 (0.9)	<0.001
Bed size							
<250	606,196 (32.0)	27,229 (27.7)	103,311 (28.5)	179,463 (32.3)	197,894 (33.8)	98,299 (33.9)	<0.001
250-499	718,066 (37.9)	38,921 (39.6)	155,422 (42.8)	213,421 (38.4)	207,479 (35.5)	102,823 (35.5)	
≥500	567,962 (30.0)	32,107 (32.7)	104,094 (28.7)	163,478 (29.4)	179,788 (30.7)	88,495 (30.6)	
Profit status							
For-profit	281,337 (14.9)	29,484 (30.0)	94,752 (26.1)	97,256 (17.5)	45,219 (7.7)	14,626 (5.1)	<0.001
Not-for-profit	1,440,853 (76.1)	53,550 (54.5)	223,580 (61.6)	409,614 (73.6)	494,496 (84.5)	259,613 (89.6)	
Other	170,034 (9.0)	15,223 (15.5)	44,495 (12.3)	49,492 (8.9)	45,446 (7.8)	15,378 (5.3)	

ADI – Area Deprivation Index. SOURCE: Authors' analysis of 100 MedPAR fee-for-service claims for beneficiaries who underwent selected operative procedures from January 1, 2014 to December 31, 2018 Note: All proportions reflect the proportion of patients for each Hospital Quality Quintile

eFigure 1. Variation in Surgical Mortality Across Hospitals and Neighborhoods

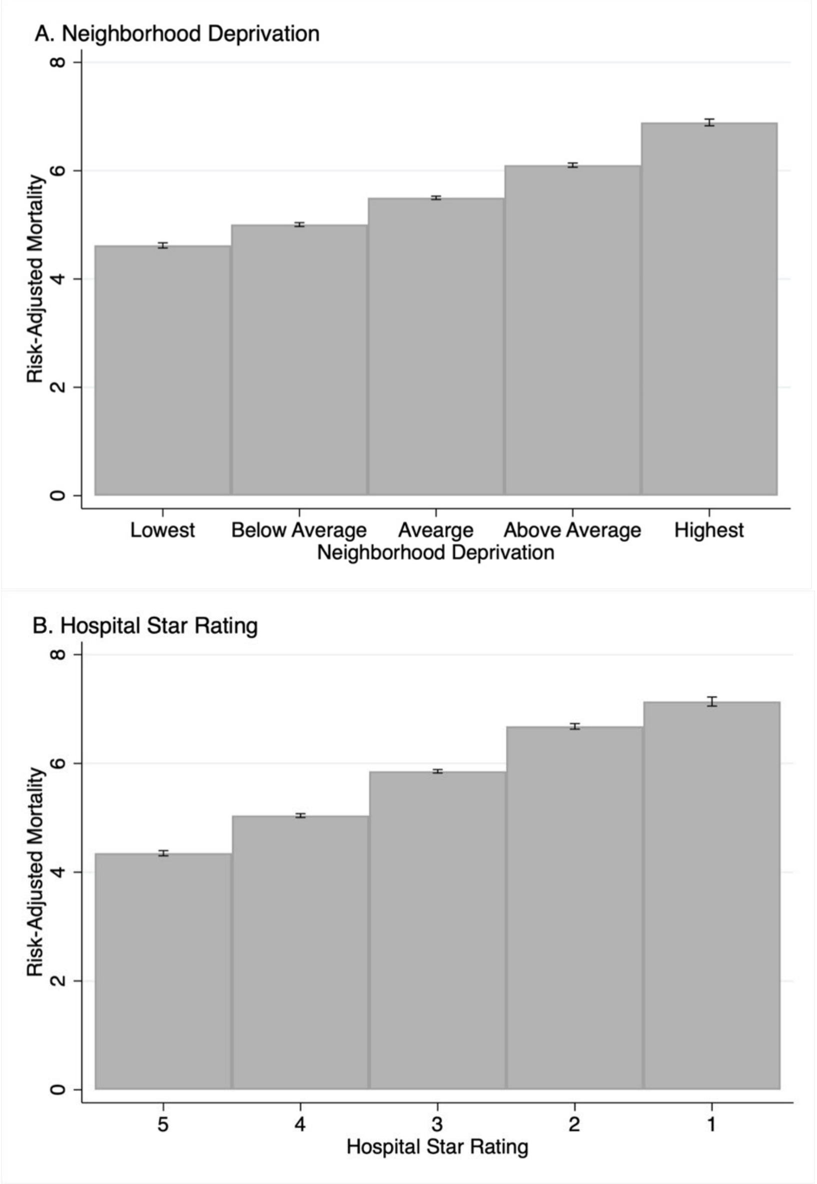

SOURCE: Authors' analysis of 100% MedPar fee-for-service claims for beneficiaries who underwent selected operative procedures from January 1, 2014 to December 31, 2018 NOTE. Risk-adjusted rates were derived from marginal means in logistic regression models. All models adjusted for patient age, sex, and 27 Elixhauser comorbidities, type of admission (i.e., elective, non-elective), overall time trends, hospital characteristics, and patients Area Deprivation Index. The Area Deprivation Index (ADI) rankings range from 1 to 100, with least disadvantaged neighborhood conditions designated by lower scores and most disadvantaged by higher scores. Examples of neighborhood-level factors incorporated within the ADI include education, employment, housing-quality, and poverty measures.

© 2023 Diaz A et al. *JAMA Network Open.*

eFigure 2. Risk Matrix of 30-day Mortality After Inpatient Surgery by Neighborhood Deprivation and Hospital Quality Among Black and White Medicare Beneficiaries

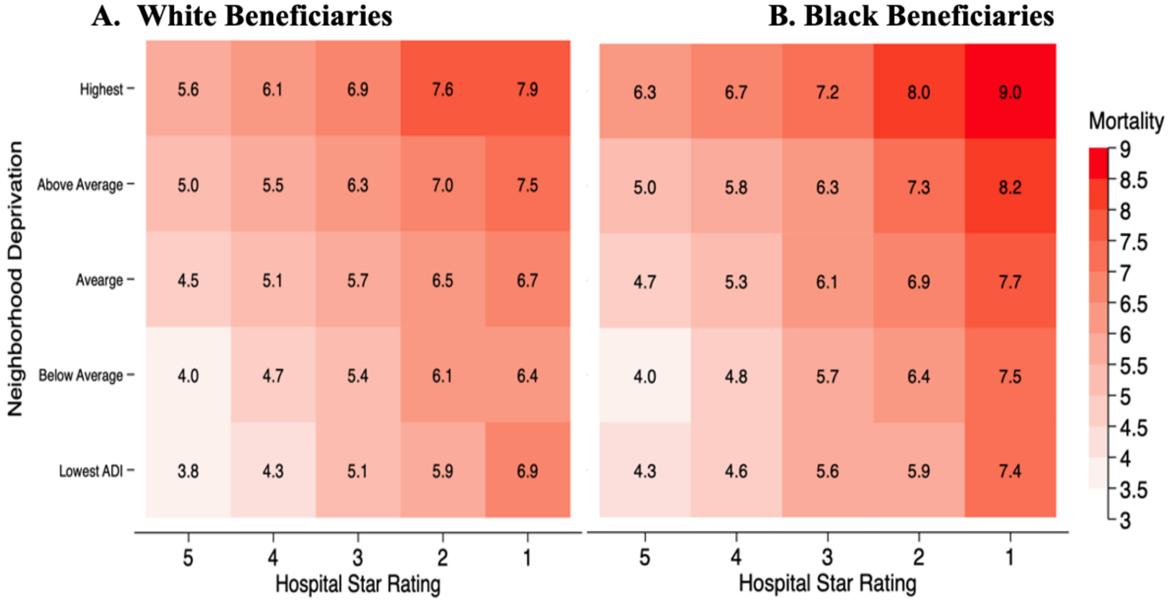

© 2023 Diaz A et al. *JAMA Network Open.*

eTable 2. Comparison of 30-Day Mortality Using Multivariable Logistic Regression Model With Clustering vs 2-Level and 3-Level Hierarchical Models

	Risk-Adjusted Odds Ratio Using Logistic Model with clustering	Risk-Adjusted Odds Ratio Using Multi-level model with Hospital Random Effects	Risk-Adjusted Odds Ratio Using Multi-level Model with Hospital and ZIP Code Random Effects
Neighborhood Deprivation			
Lowest	Ref	Ref	Ref
Below Average	1.12 (1.09-1.16)	1.09 (1.06-1.12)	1.09 (1.06-1.12)
Average	1.21 (1.17-1.25)	1.15 (1.12-1.18)	1.15 (1.12-1.18)
Above Average	1.31 (1.26-1.36)	1.22 (1.19-1.25)	1.22 (1.19-1.26)
Highest	1.39 (1.34-1.44)	1.28 (1.24-1.31)	1.28 (1.25-1.32)
Hospital Star Rating			
Five	Ref	Ref	Ref
Four	1.16 (1.09-1.22)	1.17 (1.11-1.22)	1.17 (1.11-1.23)
Three	1.33 (1.26-1.41)	1.36 (1.30-1.43)	1.38 (1.31-1.44)
Two	1.48 (1.39-1.57)	1.51 (1.44-1.59)	1.53 (1.45-1.61)
One	1.76 (1.61-1.92)	1.86 (1.73-2.00)	1.90 (1.77-2.04)

SOURCE: Authors' analysis of 100% MedPar fee-for-service claims for beneficiaries who underwent selected operative procedures from January 1, 2014 to December 31, 2018 NOTE. Risk-adjusted rates were derived from marginal means in logistic regression models. All models adjusted for patient age, sex, and 27 Elixhauser comorbidities, type of admission (i.e., elective, non-elective), year of operation, hospital characteristics, and patients Area Deprivation Index. The Area Deprivation Index (ADI) rankings range from 1 to 100, with least disadvantaged neighborhood conditions designated by lower scores and most disadvantaged by higher scores. Examples of neighborhood-level factors incorporated within the ADI include education, employment, housing-quality, and poverty measures.

© 2023 Diaz A et al. *JAMA Network Open.*

eTable 3. List of *ICD-9* and *ICD-10 Procedure* Codes

CABG

ICD9	ICD10							
3610	0210093	021209W	02100K8	02110A9	021208C	0213088	02134Z9	02124AF	0211483
3611	02100A3	02120AW	02100K9	02110AC	0212098	0213089	02134ZC	02124JF	0211493
3612	02100J3	02120JW	02100KC	02110J8	0212099	021308C	021008F	02124KF	02114A3
3613	02100K3	02120KW	02100Z8	02110J9	021209C	0213098	021009F	02124ZF	02114J3
3614	02100Z3	021248W	02100Z9	02110JC	02120A8	0213099	02100AF	021308F	02114K3
3615	0210493	021249W	02100ZC	02110K8	02120A9	021309C	02100JF	021309F	02114Z3
3616	02104A3	02124AW	0210488	02110K9	02120AC	02130A8	02100KF	02130AF	0212083
3617	02104J3	02124JW	0210489	02110KC	02120J8	02130A9	02100ZF	02130JF	0212093
3619	02104K3	02124KW	021048C	02110Z8	02120J9	02130AC	021048F	02130KF	02120A3
	02104Z3	021308W	0210498	02110Z9	02120JC	02130J8	021049F	02130ZF	02120J3
	021008W	021309W	0210499	02110ZC	02120K8	02130J9	02104AF	021348F	02120K3
	021009W	02130AW	021049C	0211488	02120K9	02130JC	02104JF	021349F	02120Z3
	02100AW	02130JW	02104A8	0211489	02120KC	02130K8	02104KF	02134AF	0212483
	02100JW	02130KW	02104A9	021148C	02120Z8	02130K9	02104ZF	02134JF	0212493
	02100KW	021348W	02104AC	0211498	02120Z9	02130KC	021108F	02134KF	02124A3
	021048W	021349W	02104J8	0211499	02120ZC	02130Z8	021109F	02134ZF	02124J3
	021049W	02134AW	02104J9	021149C	0212488	02130Z9	02110AF	0210083	02124K3
	02104AW	02134JW	02104JC	02114A8	0212489	02130ZC	02110JF	0210093	02124Z3
	02104JW	02134KW	02104K8	02114A9	021248C	0213488	02110KF	02100A3	
	02104KW	0210088	02104K9	02114AC	0212498	0213489	02110ZF	02100J3	
	021108W	0210089	02104KC	02114J8	0212499	021348C	021148F	02100K3	
	021109W	021008C	02104Z8	02114J9	021249C	0213498	021149F	02100Z3	
	02110AW	0210098	02104Z9	02114JC	02124A8	0213499	02114AF	0210483	
	02110JW	0210099	02104ZC	02114K8	02124A9	021349C	02114JF	0210493	
	02110KW	021009C	0211088	02114K9	02124AC	02134A8	02114KF	02104A3	
	021148W	02100A8	0211089	02114KC	02124J8	02134A9	02114ZF	02104J3	
	021149W	02100A9	021108C	02114Z8	02124J9	02134AC	021208F	02104K3	
	02114AW	02100AC	0211098	02114Z9	02124JC	02134J8	021209F	02104Z3	
	02114JW	02100J8	0211099	02114ZC	02124K8	02134J9	02120AF	0211083	
	02114KW	02100J9	021109C	0212088	02124K9	02134JC	02120JF	0211093	
	021208W	02100JC	02110A8	0212089	02124KC	02134K8	02120KF	02110A3	
	02124Z8	02134K9	02120ZF	02110J3	02134J3	0213483	02130J3	0213083	
	02124Z9	02134KC	021248F	02110K3	02134K3	0213493	02130K3	0213093	
	02124ZC	02134Z8	021249F	02110Z3	02134Z3	02134A3	02130Z3	02130A3	

© 2023 Diaz A et al. *JAMA Network Open.*

Colon Resection

ICD9	ICD10	
1732	0DTH4ZZ	0DBE7ZZ
1733	0DTF4ZZ	0DBF0ZZ
1734	0DTL4ZZ	0DBF3ZZ
1735	0DTG4ZZ	0DBF7ZZ
1736	0DTN4ZZ	0DBG0ZZ
1739	0DBE4ZZ	0DBG3ZZ
4571	0DBF4ZZ	0DBG7ZZ
4572	0DBG4ZZ	0DBH0ZZ
4573	0DBH4ZZ	0DBH3ZZ
4574	0DBK4ZZ	0DBH7ZZ
4575	0DBL4ZZ	0DBK0ZZ
4576	0DBM4ZZ	0DBK3ZZ
4579	0DBN4ZZ	0DBK7ZZ
4581	0DTK4ZZ	0DBL0ZZ
4582	0DTM4ZZ	0DBL3ZZ
4583	0DBE0ZZ	0DBL7ZZ
	0DBE3ZZ	0DBM0ZZ
	0DBE7ZZ	0DBM3ZZ
	0DTH0ZZ	0DBM7ZZ
	0DTH7ZZ	0DBN0ZZ
	0DTF0ZZ	0DBN3ZZ
	0DTF7ZZ	0DBN7ZZ
	0DTK0ZZ	0DTK0ZZ
	0DTL0ZZ	0DTK7ZZ
	0DTL7ZZ	0DTM0ZZ
	0DTG0ZZ	0DTM7ZZ
	0DTG7ZZ	0DTE4ZZ
	0DTN0ZZ	0DTE0ZZ
	0DTN7ZZ	0DTE7ZZ
	0DBE0ZZ	0DBE3ZZ

© 2023 Diaz A et al. *JAMA Network Open.*

Incisional Hernia

ICD9	ICD10
5351	0WQF0ZZ
5359	0WQF3ZZ
5361	0WQF4ZZ
5362	0WQF0ZZ
5363	0WQF3ZZ
5369	0WQF4ZZ
	0WUF07Z
	0WUF0JZ
	0WUF0KZ
	0WUF47Z
	0WUF4JZ
	0WUF4KZ
	0WUF47Z
	0WUF4JZ
	0WUF4KZ
	0WUF07Z
	0WUF0JZ
	0WUF0KZ

Cholecystectomy

ICD9	ICD10
5121	0F540ZZ
5122	0F543ZZ
5123	0FB40ZZ
5124	0FB43ZZ
	0FT40ZZ
	0FT44ZZ
	0F544ZZ
	0FB44ZZ

Appendectomy

ICD9	ICD10
4701	0DTJ4ZZ
4709	0DTJ0ZZ
4711	0DTJ7ZZ
4719	0DTJ4ZZ
	0DTJ0ZZ
	0DTJ7ZZ

© 2023 Diaz A et al. *JAMA Network Open.*
