# Supplementary material for: Association of Hospital Quality and Neighborhood Deprivation With Mortality After Inpatient Surgery Among Medicare Beneficiaries
Source: JAMA Netw Open. 2023 Jan 30;6(1):e2253620. doi: 10.1001/jamanetworkopen.2022.53620 (PMC9887494; doi:10.1001/jamanetworkopen.2022.53620)
Supplement: Supplement 2. — Data Sharing Statement [file jamanetwopen-e2253620-s002.pdf]

Data Sharing Statement

Diaz. Association of Hospital Quality and Neighborhood Deprivation With Mortality After Inpatient Surgery Among Medicare Beneficiaries. *JAMA Netw Open*. Published January 30, 2023. doi:10.1001/jamanetworkopen.2022.53620

Data
Data available: No

Additional Information
Explanation for why data not available: Unable to publicly share data due to DUA. Data available upon request
